# ‘Fast’ and ‘slow’ water handling strategies explain pinyon pine decline and juniper expansion

**DOI:** 10.1007/s00442-026-05929-y

**Published:** 2026-07-20

**Authors:** Faraz Rehman, Andrew Kulmatiski, Lillian Gordon, Ryan Sandfort, Kyle A Palmquist

**Affiliations:** 1 Department of Wildland Resources and the Ecology Center, Utah State University, Logan, UT USA; 2 Biological Sciences, Marshall University, Huntington, WV USA

**Keywords:** Ecohydrology, Isotope, Niche partitioning, Pinyon-juniper, Soil water, Tracer

## Abstract

Semiarid forests in the western USA have undergone large-scale shifts in dominance due to decreasing pinyon pine abundance and increasing juniper abundance. This shift has been attributed to fast water loss in aboveground pinyon tissues during a long-term drought. Water loss, or ‘outputs’, however, are only a part of a plant’s water budget. Water inputs, or root water uptake, are also important, but rarely well understood. We used depth-controlled tracer experiments, root water potentials, and soil water flow models to estimate pinyon and juniper inputs (root water uptake) and stomatal conductance as a proxy for water outputs. Measurements were made over two years at three sites that crossed an aridity gradient, Utah, USA. Pinyon roots were good at ‘following’ water through the soil, but not good at absorbing water from dry soil. Pinyon tracer uptake profiles (1–90 cm) changed more from month to month than juniper (coefficient of variation = 0.25 ± 0.02 and 0.16 ± 0.02, respectively) in ways that provided 12% more water over time. However, because pinyon roots had higher water potentials, the net water uptake advantage for pinyon was smaller: over six sampling dates, pinyon absorbed 2% more tracer than juniper. Aboveground, pinyon stomatal conductance was 25% greater than juniper. Results indicate that pinyon both absorbs and releases water faster than juniper. These ‘fast’ aboveground and belowground strategies place pinyon at higher risk of rapid transitions from growth to mortality than previously thought from aboveground research alone and suggests continued pinyon mortality in the future.

**Supplementary Information:**

The online version contains supplementary material available at 10.1007/s00442-026-05929-y.

## Introduction

Pinyon and juniper woodlands cover 40 million hectares across the western United States (USA) and play an important role in shaping landscape diversity, yet these woodlands are undergoing rapid changes, with pinyon decreasing and juniper increasing (Breshears et al. 2005; Romme et al. 2009; Squires and Gaur 2018; Shriver et al. 2022). Juniper expansion is one of the largest contributors to woody encroachment in the western USA, with juniper often doubling or tripling cover in sagebrush-steppe systems in the past century (Miller et al. 2008; Van Auken 2009). Consequences of juniper expansion include decreased forage production, soil erosion, and increased fire intensity (Romme et al. 2009; Miller et al. 2019; Shinneman et al. 2023). Consequences of pinyon decline include decreases in ecologically and culturally important resources (e.g., pine nuts).

Given their extent and rate of change, there has been interest in understanding why pinyon is declining and juniper is expanding (Shriver et al. 2022, 2025). Juniper expansion has been linked to fire suppression, land-use change, rising CO₂, and a conservative water use strategy (Miller and Rose 1999; West et al. 2007; Redmond et al. 2023). Pinyon decline has been attributed to long-term drought conditions (Ogle et al. 2000; Fair et al. 2018; Flake and Weisberg 2019). More specifically, increasing temperatures and aridity have been suggested to increase physiological stress on pinyon by limiting both water uptake and use strategies (Floyd et al. 2009; Redmond et al. 2023). Pinyon has higher photosynthetic rates but lower water-use efficiency than juniper (Linton et al. 1998; Peterman et al. 2013), while juniper is more drought tolerant, maintaining lower water potentials and exhibiting lower vulnerability to cavitation (Williams and Ehleringer 2000; Guo et al. 2024). Pinyon is more isohydric, closing its stomata early under dry conditions, while juniper is more anisohydric, maintaining stomatal opening during dry conditions, these different strategies may allow for coexistence of pinyon and juniper because pinyon can grow quickly when water is available and juniper can continue to grow slowly even when water is less available (Lajtha and Barnes 1991; West et al. 2008; Kraklow et al. 2025).

While aboveground water use strategies are likely to be important to pinyon and juniper drought responses, it is also likely that belowground water uptake strategies are important, but relatively little is known about how these plant species absorb their water (Hartsell et al. 2020; Shinneman et al. 2023; Noel et al. 2023). This is an important knowledge gap because plants with root systems that can absorb more water tend to be more abundant in water-limited ecosystems (Forero and Kulmatiski 2025; Kulmatiski et al. 2020a; Kulmatiski and Beard 2022). Whether pinyon and juniper growth is determined by belowground water uptake or aboveground water use has important implications for predicting their abundances over time (Linton et al. 1998; West et al. 2008; Heras et al. 2011; Peterman et al. 2013). For example, if belowground water uptake is important, then it is possible that fewer but larger precipitation events would benefit species with deeper roots because larger precipitation events will allow water to percolate deeper into the soil profile (Schwinning et al. 2004; Eggemeyer et al. 2008). However, if aboveground water use (total amount of water used, cavitation potential, water use efficiency) is important, then fewer but larger precipitation events may benefit species with more conservative aboveground water use that allows plants to survive between precipitation events (McDowell et al. 2008; Nippert and Knapp 2007; Schwinning and Ehleringer 2001).

Water uptake has long been inferred from root biomass distributions rather than direct measurements of water uptake (Ward et al. 2013; Silvertown et al. 2015). However, root biomass can be a poor indicator of water uptake because, for example, a large portion of root biomass may be structural and not directly involved in water absorption (McCully 1999; Comas et al. 2013; Cai et al. 2018). Even fine roots that are able to absorb soil water (i.e., active roots) cannot absorb water from dry soils (i.e., soils with water potentials that are lower than the water potential of the roots; Zarebanadkouki et al. 2019; Cai et al. 2022; Holz et al. 2024). Natural abundance stable isotope techniques provide more direct measurements of water uptake, but provide coarse, point-in-time estimates, typically cannot estimate water uptake below evaporation fronts (~ 20–50 cm), and are susceptible to isotope fractionation error (Dawson and Ehleringer 1998; Rothfuss and Javaux 2017). Though more difficult to perform, depth-controlled tracer techniques, developed in the past 15 years, have addressed many of these problems (Beyer et al. 2018; Marshall et al. 2020; Kulmatiski 2024; Jung et al. 2025). When combined with soil water flow models, tracer/modeling techniques can provide detailed estimates of water uptake by different species in the field over time and under a wide range of soil and climate conditions (Rasmussen and Kulmatiski, 2021; Rothfuss and Javaux 2017).

Recent studies using combined tracer/modeling approaches have revealed that plants with roots that can absorb the most water tend to be most abundant in water limited systems (Mazzacavallo and Kulmatiski 2015; Kulmatiski et al. 2024). Even small differences in active root distributions can have biologically important effects on plant abundance on the landscape (Casper and Jackson 1997; Kulmatiski et al. 2020b). Recently, it has also been suggested that slightly deeper and more flexible active root distributions tend to absorb more water than shallower and more static rooting distribution (Schenk and Jackson 2002; White et al. 2013; Kulmatiski 2024). However, these ideas have developed from relatively few depth-controlled tracer studies and need further testing particularly in more arid conditions than have been tested to date, to determine how water uptake strategies affect plant growth.

Our overarching goal was to quantify belowground water uptake and aboveground water use strategies of pinyon and juniper to understand the processes behind pinyon decline and juniper expansion. We hypothesized that pinyon is declining and juniper is expanding because pinyon has shallower and more static rooting patterns that provide less water than juniper under drying conditions, while juniper has deeper and more flexible rooting distributions. From this hypothesis, we predicted that relative to juniper, pinyon will have shallow root distributions that vary less over time and absorb less water. To test these ideas, we injected deuterium oxide (D₂O) to five to six soil depths at six times over two years in three sites that varied in aridity (~ 0.15 to 0.40). To estimate water uptake between injection dates, we used a soil water flow model. Because juniper can maintain very low water potentials, we also measured pinyon and juniper root water potentials and used these values in a second soil water flow simulation to estimate how much more water juniper roots can absorb due to their lower water potentials. To assess aboveground water use, we measured leaf water potentials and stomatal conductance.

## Methods

**Fig. 1:**
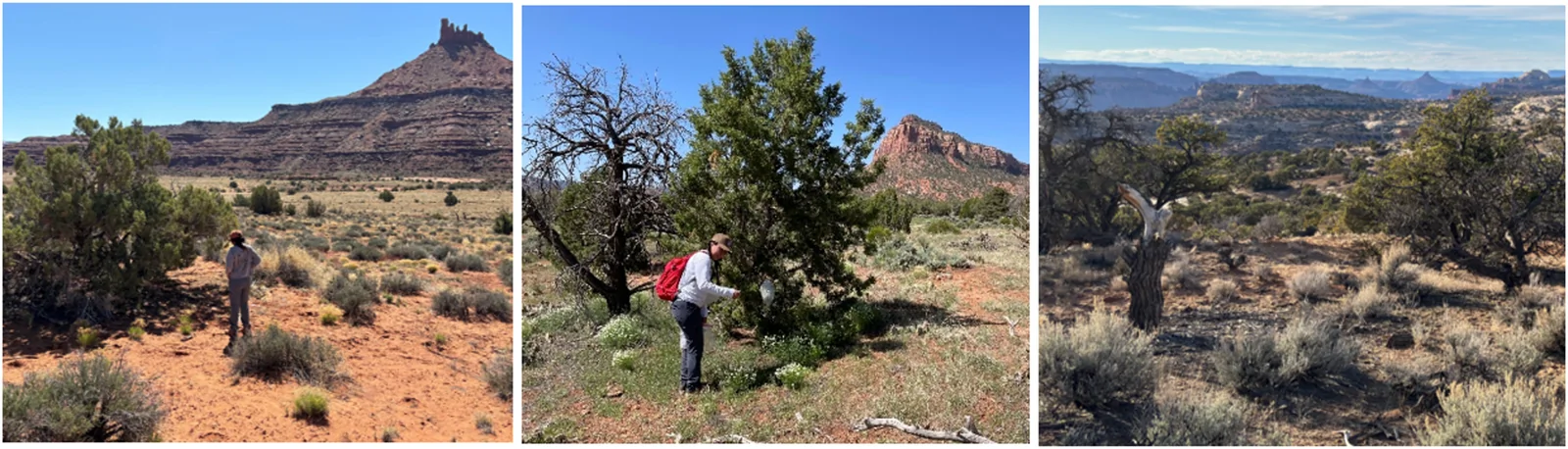
Photographs of the (**a**) Low, (**b**) Mid, and (**c**) High study sites. Site details listed in Table 1

**Study Site.** Research was conducted near the Canyonlands Research Center, Monticello, Utah, USA (38° 4’ 15.6” N, 109° 33’ 54” W) during the 2024 and 2025 growing seasons (Fig. 1). Three study sites, each approximately 13 ha, were established along an elevation and aridity gradient (aridity = annual precipitation / potential evapotranspiration; Table 1). The low-elevation site (Low site) represents the driest conditions where juniper can occur, and only juniper is present. The mid-elevation site (Mid site) represents the driest conditions where pinyon can occur, and both pinyon and juniper are present. The high-elevation site (High site) occurs under climatic conditions suitable for both species. Soils at all sites were sandy loams (Table 1). At the Low site, for which a meteorological station is closest, the 2024 growing season (October-October) was slightly wetter than the long term average (197 mm) while the 2024 growing season was drier than the long term average (143 mm). Temperatures were the same both years (13.3 °C).

**Table 1:** Site characteristics including elevation (elev), aridity index (AI), mean annual temperature (MAT) and precipitation (MAP; 800 m resolution; 30-year averages; PRISM Climate Group, Oregon State University, https://prism.oregonstate.edu), Tree and Shrub ground cover, soil texture, and bare ground cover at Low, Mid, and High sites

Site	Elev (m)	AI (%)	MAT (°C)	MAP (mm)	Tree cover (%)	Shrub cover (%)	Sand (%)	Silt (%)	Clay (%)	Bare ground (%)
Low	1517	15	12.3	186	13	79	73.9	16.3	9.7	8
Mid	1805	27	11.1	236	71	24	61.1	22.7	16.2	5
High	2031	38	10.6	267	63	31	73.0	17.2	9.7	6

Tree cover at each site was estimated using a point-intercept method applied to visual interpretation of high-resolution satellite imagery (≤ 1 m spatial resolution; Google Earth Pro 2024; Table 1). A regular grid of 1,000 points extending 2 km laterally and ± 200 m in elevation from the center of each site was overlaid on high-resolution aerial imagery. At each grid point, vegetation was visually classified as tree or non-tree based on visual inspection – pinyon and juniper canopies are darker than shrub and grass canopies (Fig. 1). Tree cover was higher at the Mid and High sites than at the Low site (Table 1). Non-tree ground cover was estimated visually for each species present as a value between 1 and 100% with 1% precision in each of 30, 1 m² plots located at 10 m intervals along three randomly located 100 m transects in each site and season. The low site was dominated by snakeweed (*Gutierrezia sarothrae*), Indian ricegrass (*Achnatherum hymenoides*), blackbrush (*Coleogyne ramosissima*), ephedra (*Ephedra viridis*), galleta grass (*Pleuraphis jamesii*) and Utah juniper (*Juniperus osteosperma*). The Mid site was dominated by galleta grass (*Pleuraphis jamesii*), sagebrush (*A. tridentata*), snakeweed (*G. sarothrae*), ricegrass (*A. hymenoides*), plains pricklypear cactus (*Opuntia polycantha*), saltbush (*Atriplex canescens*), Utah juniper (*Juniperus osteosperma*), and pinyon pine (*Pinus edulis*). The High site was dominated by galleta grass (*P. jamesii*), sagebrush (*A. tridentata*), Utah juniper (*J. osteosperma*), pinyon pine (*P. edulis*), Indian ricegrass (*A. hymenoides*), and plains pricklypear cactus (*O. polycantha*). Species nomenclature follows the PLANTS Database (United States Department of Agriculture, Natural Resources Conservation Service 2024).

**Tracer Experiments.** To quantify seasonal vertical patterns of root water uptake, we conducted tracer injections at five to six soil depths (1, 5, 15, 30, 60 and 90 cm) across the growing season. Injections were performed during early (March), mid (April), and late (May) seasons in both 2024 and 2025. In 2024, three pinyon and three juniper individuals (diameter at breast height > 5 cm), separated by at least 5 m, were selected to represent each month × depth treatment level at sites where each species was present (*n* = 3 months × 5 depths × 3 replicates × 2 species at Mid and High sites; *n* = 3 months x 5 depths x 3 replicates x 1 species at the Low site, total *n* ≈ 225 trees). In 2025, sampling was expanded to include additional replicates and a deeper soil injection depth (90 cm). Six replicates were collected at 1, 5, 15, and 30 cm, five replicates at 60 cm, and four replicates at 90 cm (*n* = 3 months × 6 depths × 2 species at the Mid and High sites; *n* = 3 months × 6 depths × 1 species at the Low site × 4–6 replicates ≈ 495 trees). By the end of the study, 720 trees were sampled, representing most of the pinyon and more than half of the juniper population within each study site.

At the time of tracer injection, 16 pilot holes were made in a grid (points separated by 50 cm) around the bole of the target tree. Pilot holes were created using a hammer drill (Hilti-TE60; Hilti USA) with 1 cm-wide bit to the target depth. To be clear, each tree represented one site × month × depth × replicate combination, so all pilot holes around a tree were drilled to one depth (e.g., 60 cm). Custom-made 5 mL syringes with 16 gauge thin-walled hypodermic tubing (Vita Needle, MA, USA) were used to inject 1 mL 70% D_2_O to the bottom of each pilot hole. Tracer injections were followed by two separate, 1 mL tap-water injections used to clear tracer out of the hypodermic tubing. Injections were assumed to act as tracers and not resource pulses that stimulated plant growth because the 48 ml of tracer and rinse water added to the roughly 4 m^2^ injection area represented 0.012 mm of water.

One day after injections, roughly 5 L of branch and leaf volume were enclosed in transparent 75 μm-thick polyethylene bags (3 mil boot covers; Uline Inc. WI, USA) and sealed with zip ties to collect transpired water (Kulmatiski and Forero 2021). One to three bags were placed on each tree depending on tree size. After 48 h, transpired water was collected and placed in 2 mL vials. Pipettes used to collect water samples were triple rinsed with tap water between each sample. Samples were placed in a cooler within two hours in the field, then refrigerated until analysis. Seven control samples were collected from random trees that received no tracer for each species.

**Soil Samples.** Three soil cores (5 cm diameter, 75 cm depth) were collected on each sampling date and split into 15 cm increments. Soil samples were dried to a constant weight at 65 °C, passed through a 2-mm sieve. Samples were used to estimate soil bulk density, soil texture, and gravimetric water content, which were used in soil water flow model analyses (see below; Table S1).

**Stomatal conductance.** Stomatal conductance (mmol m⁻² s⁻¹) was measured for each target tree using a steady-state porometer (METER SC-1 Leaf Porometer; Meter Group, Inc., Pullman, WA, USA) during each season (early, mid, late) in both 2024 and 2025. Measurements were taken on attached leaves during consistently sunny days for at least three trees of each species during each tracer injection campaign. Tops and bottoms of leaves were not distinguished on the needle-like leaves of the target species, so stomatal conductance represents the average from the tops and bottoms of leaves.

**Water potential.** Leaf and root water potential was measured for both pinyon and juniper. Samples were collected on September 5, 2025 between 1200 and 1400 h to assess soil-to-root water potential gradients under active transpiration (Turner 1988). Fine roots (~ 1–2 mm diameter) were collected under dense pinyon or juniper canopies where other plant species were absent. Root and leaf water potential samples were collected again in November 20, 2025 between 1400 and 1600 h. All leaf and root samples were wrapped in aluminum foil, sealed in plastic bags and placed in a cooler until measurement (Tomasella et al. 2023). Prior to measurement, leaf surfaces were lightly abraded with sandpaper to remove the waxy cuticle without damaging the leaf and to ensure proper contact with the chamber. All water potential measurements were made using the chilled-mirror technique within two hours of sample collection (WP4C Water Potential Meter; Meter Group, Inc., Pullman, WA, USA).

**Data Analysis.** Collected transpired water samples were analyzed for deuterium (²H) and oxygen-18 (¹⁸O) concentrations using a cavity ring-down spectrometer (L2120i; Picarro Inc., California, USA). To limit carryover of high deuterium (D₂O) concentrations between samples, tap water samples were run between each experimental sample. Further, each sample was analyzed five times by the instrument, and only the final instrument sub-replicate measurement was recorded. Deuterium concentrations (delta notation) were then adjusted using a linear slope and intercept calibration developed from three known standards in each tray of 23 samples. To control for natural evaporative enrichment, deuterium excess (d_excess_) values were calculated as follows:

$$\:{d}_{\mathrm{excess}}={\delta\:}^{2}\mathrm{H}-\left[8\times\:\left({\delta\:}^{18}O\right)+10\right]$$

where δ^18^O is the oxygen-18 value for each sample, and the slope and intercept reflect the Global Meteoric Water Line (GMWL). To minimize false positive tracer detection, d_excess_ values for control samples for pinyon (49‰) and juniper (55‰) were subtracted from all experimental sample values and adjusted values < 0 were set to zero to avoid interpreting background variation (Kulmatiski et al. 2010; Beyer et al. 2016).

Water tracer uptake values are typically normalized into the proportion of tracer uptake with depth (Kulmatiski et al. 2010; Mazzacavallo and Kulmatiski 2015). This is done to allow a direct comparison of tracer uptake in small plants to tracer uptake in large plants (e.g., grasses and trees). However, it was not necessary to convert tracer concentrations into proportions in this study because pinyon and juniper are roughly the same size. Using d_excess_ values instead of proportion tracer uptake values provides the advantage that d_excess_ values allow a quantitative estimate of total water uptake as well as indicating variation in uptake with soil depth. For comparison to other studies, normalized (proportion) tracer uptake values are reported in supplementary materials (Figs. S2 and S3).

To test if one species absorbed more tracer across depths than the other, we used a Welch two-sample t-test. To describe patterns of tracer uptake by soil depth and plant species, generalized additive mixed models were used (GAMMs; Wood 2004). The 2024 and 2025 datasets were modeled using four or five knots, respectively to describe the five and six injection depths. A Guassian distribution with logit link were used (Kulmatiski et al. 2017). For each season*site*species combination, two models were fit: (1) a global model with depth as the only factor and (2) a species-specific model with depth and ‘species’ as two factors. Lower AIC statistics indicated a balance of goodness-of-fit against parsimony (Wood 2004). If the ‘species-specific’ model has a lower AIC than the ‘global’ model, this suggests that tracer uptake varies between plant species. All models were run in R (R Core Team 2018) using the mgcv package.

To assess the directional hypothesis that pinyon uses ‘faster’ root foraging we tested the prediction that pinyon exhibits greater variability in d_excess_ than juniper across sampling months. To do this, differences in the coefficient of variation (CV) of d_excess_ by depth and species were tested using a one-tailed Wilcoxon signed-rank test. The Wilcoxon test examined whether the median of depth-wise CV differences (Pinyon CV minus Juniper CV) were significantly greater than zero or not. In other words, CV values for each soil depth were used as replicates to test if pinyon demonstrated greater variation in uptake over time across soil depths relative to juniper.

**Water Uptake.** Tracer uptake was used to describe active root distributions at the time of injections. To estimate water uptake by those roots over time, a soil water flow model was used. The Hydrus 1D water flow model replicates hydrological processes including water interception by the plant canopy, infiltration, evaporation, and percolation, as well as extraction by a given root system (Šimůnek et al. 2016; Radcliffe and Simunek 2018). We used site-specific meteorological inputs, including precipitation and measured soil properties. The model was initialized using an ecosystem-level active root distribution estimated from tracer uptake by all species at the low site (measured as part of a separate experiment; Kulmatiski and Beard 2022). Soil hydraulic parameters were selected using inverse modeling and weekly observed soil moisture at 10, 25, and 50 cm between October 2023 and March 2024 at the Low site (Table S1). Inverse modeling was used sequentially to select hydraulic conductivity, l, alpha, then n values. Next, inverse models were used sequentially to select the best combination of these factors one at a time for the five soil materials represented in the model. Final parameter selection indicated that the model captures general patterns of soil water flow at the community level over time (Fig. S1). Model parameters selected in this inverse model fitting exercise were applied to the other sampling dates and sites (observed soil moisture data was not available for the other sites and the other sites have similar soil textures). Model parameters needed to reproduce these analyses are described in supplementary materials Table S1. Isotope-enabled modeling options in Hydrus were not used because we did not follow our injected tracers through the soil, so validation of this model was not possible. We did not follow the tracer in the soils because it is difficult to relocate the small injection points, particularly in deeper soil depths.

We then used our tracer uptake data to parse this validated, community-level water uptake data into pinyon vs. juniper water uptake. For example, Hydrus may estimate that 10 mm of water is absorbed from the top 10 cm of soil in May. If pinyon and juniper tracer concentrations were 80‰ and 20‰ in the top 10 cm, respectively, then pinyon was assumed to absorb 80% of water in the top 10 cm and juniper was assumed to absorb 20%. Thus, pinyon would be estimated to absorb 8 mm and juniper 2 mm of water from the top 10 cm of soil in May.

Because juniper has been reported to have lower root and leaf water potentials than pinyon, we used a second Hydrus simulation to estimate root water uptake for a species with either a high (i.e., -2.5 MPa) or low (i.e., -4.5 MPa) root water potential. We assume pinyon water uptake is represented by water uptake at -2.5 MPa and juniper water uptake is represented by water uptake at -4.5 MPa. These values represented the minimum water potential we observed for each species in our samples and are consistent with published values (Plaut et al. 2012). More specifically, the pressure head at which root water uptake ceases (P3) was assigned as -2.5 MPa and water was partitioned to pinyon and juniper (Feddes et al. 1976; Plaut et al. 2012). Next, the model was run with a P3 value of -4.5 MPa. Any additional water uptake in the − 4.5 MPa model was assumed to be absorbed by juniper.

**Stomatal Conductance.** Stomatal conductance (g_s) data from 2024 to 2025 were analyzed to test the effects of species, site, and season. Because log transformation cannot accommodate zero values, we added a small constant (ε) to all conductance observations prior to transformation, where ε was defined as half the smallest non-zero conductance value in the dataset (i.e., 0.9 mmol m^− 2^ sec^− 1^). We then analyzed log-transformed conductance as log(g_s + ε). We analyzed each year separately, because each year represented wet and dry conditions. For each year, we fit a linear model$\:\mathrm{log\_conductance}\sim\:\mathrm{Species}\times\:\mathrm{Season}\times\:\mathrm{Site},$.

and tested, the significance of main effects and interactions using Type III sums of squares (car::Anova, type = 3). To quantify species differences within each year, we used post hoc pairwise comparisons using estimated marginal means (emmeans package in R). Species differences were expressed as pinyon-to-juniper ratios with 95% confidence intervals (Lenth and Piaskowski 2017).

**Water Potential.** Leaf and root water potential were calculated as the mean of daytime measurements for each species and tissue type. Mean and standard error (SE) of Ψ_leaf were calculated for each species using the dplyr package in R (Wickham et al. 2014). Distributions of water potential values were examined and found to be normal. However, an F-test showed unequal variances between species. Therefore, to test differences in mean water potential between species, we performed a two-sample t-test assuming unequal variances (t.test (Mpa ~ Species), with significance set at *p* < 0.05.

## Results

**Tracer uptake.** Across all sampling dates and sites, we analyzed 281 pinyon and 421 juniper transpiration water samples. Of these 702 samples, 655 demonstrated deuterium concentrations greater than control values (-49‰ for pinyon and − 55‰ for juniper). These control values represent baseline (non-enriched) conditions, and tracer detection was based on enrichment relative to this background. A more conservative cutoff of, for example, the mean control value plus two standard deviations would be likely to remove samples from the dataset that clearly did not absorb tracer, thereby biasing the dataset toward enriched samples. Mean deuterium concentrations (δ²H) were greater in pinyon (144 ± 13.8‰ SE) than in juniper (122 ± 4.74‰; Welch’s *t*_*6,479*_ =2.63, *p* < 0.009). Total tracer uptake was generally greater in wet conditions. The sum of tracer uptake across depths was 9690‰ in the wet year and 2553‰ in the dry year; 6541‰ at the High site and 5702‰ in the Mid site; 7956‰ in the early season, 5818‰ in the mid season, and 4592‰ in the late season.

**Fig. 2:**
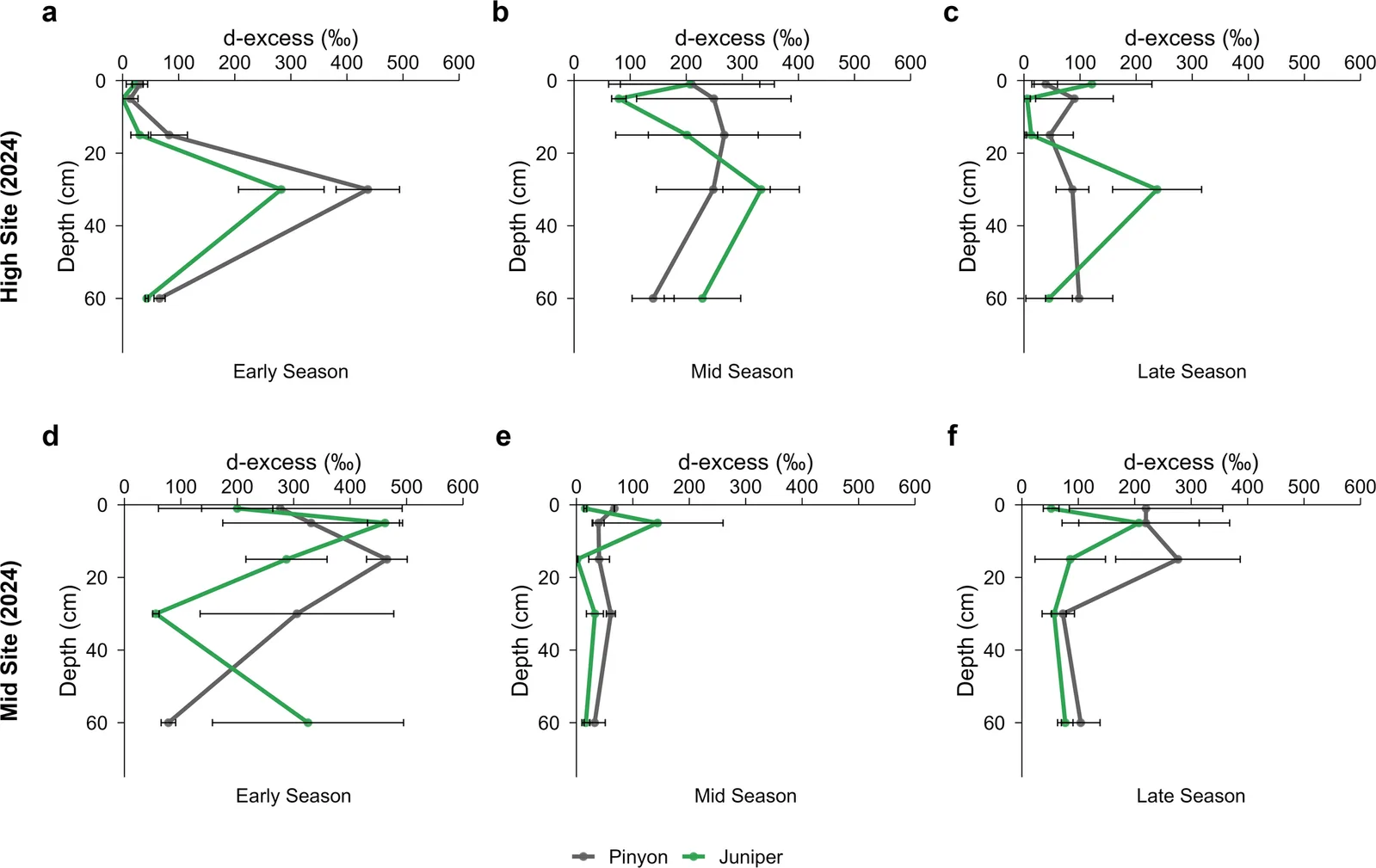
Hydrologic tracer concentrations (d_excess_ in ‰) measured in transpired water from pinyon and juniper during the wet year (2024) at the High site (panels **a–c**) and the Mid site (panels **d–f**) across early (**a**, **d**), mid (**b**, **e**), and late seasons (**c**, **f**). Values represent mean d_excess_ ± SE. Error derived from 3–6 replicate trees for each site*month*depth combination

**Fig. 3:**
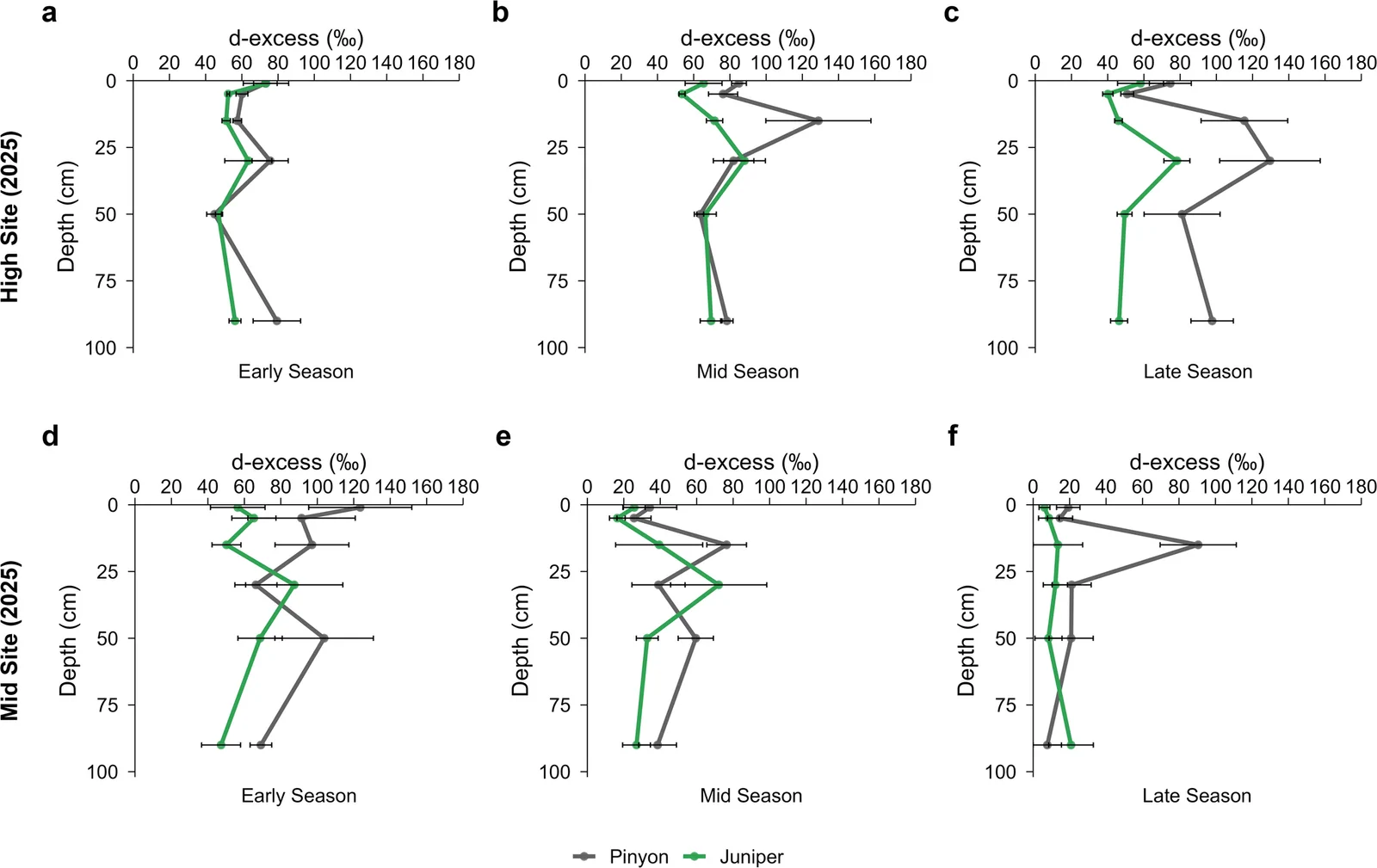
Hydrologic tracer concentrations (d_excess_ in ‰) measured in transpired water from pinyon during the dry year (2025) at the High site (panels **a–c**) and the Mid site (panels **d–f**) early (**a**, **d**), mid (**b**, **e**), and late seasons (**c**, **f**). Values represent mean d_excess_ ± SE. Error derived from 3–6 replicate trees for each site*month*depth combination

There was more support for models that separated pinyon and juniper tracer uptake profiles than for models that grouped pinyon and juniper tracer uptake profiles (Figs. 2 and 3; Table 2; Fig S2 and S3). In other words, pinyon and juniper had different depth dependent water uptake strategies. More specifically, for 11 of 12 site×month comparisons, there was equal or more support for models that separated pinyon and juniper values by depth than for models that grouped pinyon and juniper values by depth (Table 2). The mean depth of tracer uptake can be an indicator of root profile differences, but there was no difference in the mean depth of tracer uptake between pinyon (25 ± 2 cm) and juniper (28 ± 2 cm; t_1,22_ = 0.75, *p* = 0.47) across sites and months.

**Table 2:** Akaike Information Criterion (AIC) comparison of Gaussian generalized additive models testing shared (global) versus species-specific (factor-smooth) depth responses for pinyon and juniper across seasons and sites in 2024 (wet year) and 2025 (dry year). Model support was evaluated using ΔAIC (Global − Species), where values > 2 indicate meaningful support for the species-specific model, values between 0–2 indicate weak support, and values ≤ 0 indicate no improvement over the global model. Lower AIC values indicate better model fit and are indicated in bold letters

Site	Season	2024			2025			df (Global)	df (Species)
		AIC (Global)	AIC (Species)	ΔAIC	AIC (Global)	AIC (Species)	ΔAIC		
Mid	Early	281	281	0	674	**670**	**4**	3	2
Mid	Mid	314	**312**	**2**	602	602	0	3	2
Mid	Late	368	369	−1	619	**606**	**12**	3	2
High	Early	371	370	1	529	529	0	4	8
High	Mid	343	**341**	**2**	566	565	1	3	2
High	Late	385	384	1	556	**548**	**8**	4	4

**Fig. 4:**
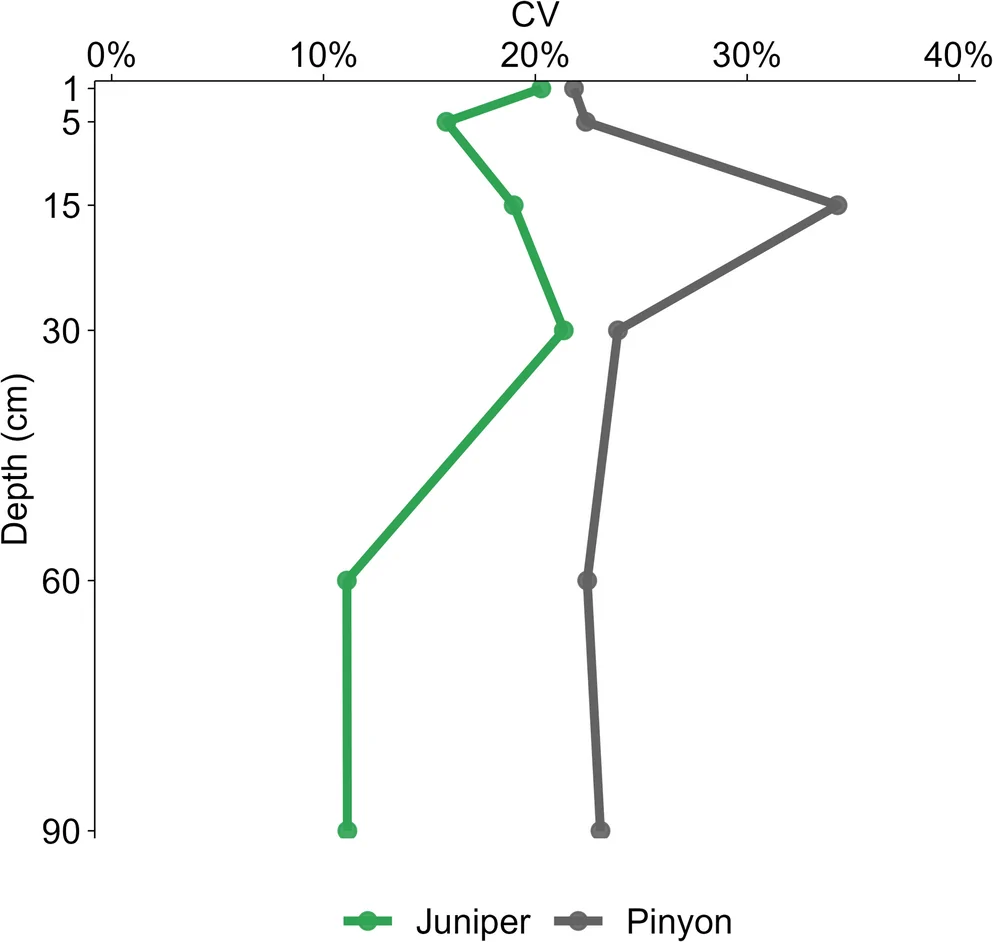
Depth-specific variation in tracer uptake (CV %) over time for pinyon and juniper at two study sites. Values represent the CV across 12 sampling date*site combinations

Pinyon uptake was more variable over time with peaks at every depth during different months (Figs. 2 and 3). Greater variability in pinyon uptake was reflected in greater CV values for pinyon than juniper (Fig. 4; t_1,5_ = 3.64, *p* = 0.007). Across depths, the CV of tracer uptake was 25% for pinyon and 16% for juniper (Fig. 4).

**Water uptake.** Water uptake between sampling events was estimated using the soil water flow model Hydrus, but these values were not tested statistically because they result from combining the tracer uptake data (described above) with deterministic output of the soil water flow model. In the wet year (2024), total water uptake was greater at the High site (10.1 cm) than at the Mid site (8.76 cm). At the Mid site, pinyon absorbed (4.55 cm) approximately 8% more total water than juniper (4.21 cm), while at the High site pinyon absorbed (5.47 cm) approximately 16.8% more water than juniper (4.68 cm). In the dry year (2025), total water uptake was less across sites and species. At the Mid site, pinyon absorbed (1.18 cm) approximately 21% more total water than juniper, whereas at the High site total water uptake was similar between species, with pinyon absorbing 1.23 cm, only about 1.8% more water than juniper (1.21 cm).

In a second Hydrus simulation designed to estimate the effect of water uptake by a species with lower water potentials (i.e., juniper with minimum water potentials of -4.5 MPa instead of -2.5 MPa), root water uptake increased 12% (0.97 cm). We assume this water was available to juniper and not pinyon because of juniper’s ability to absorb water at lower water potentials than pinyon (Robinson et al. 2010; Plaut et al. 2012).

**Fig. 5:**
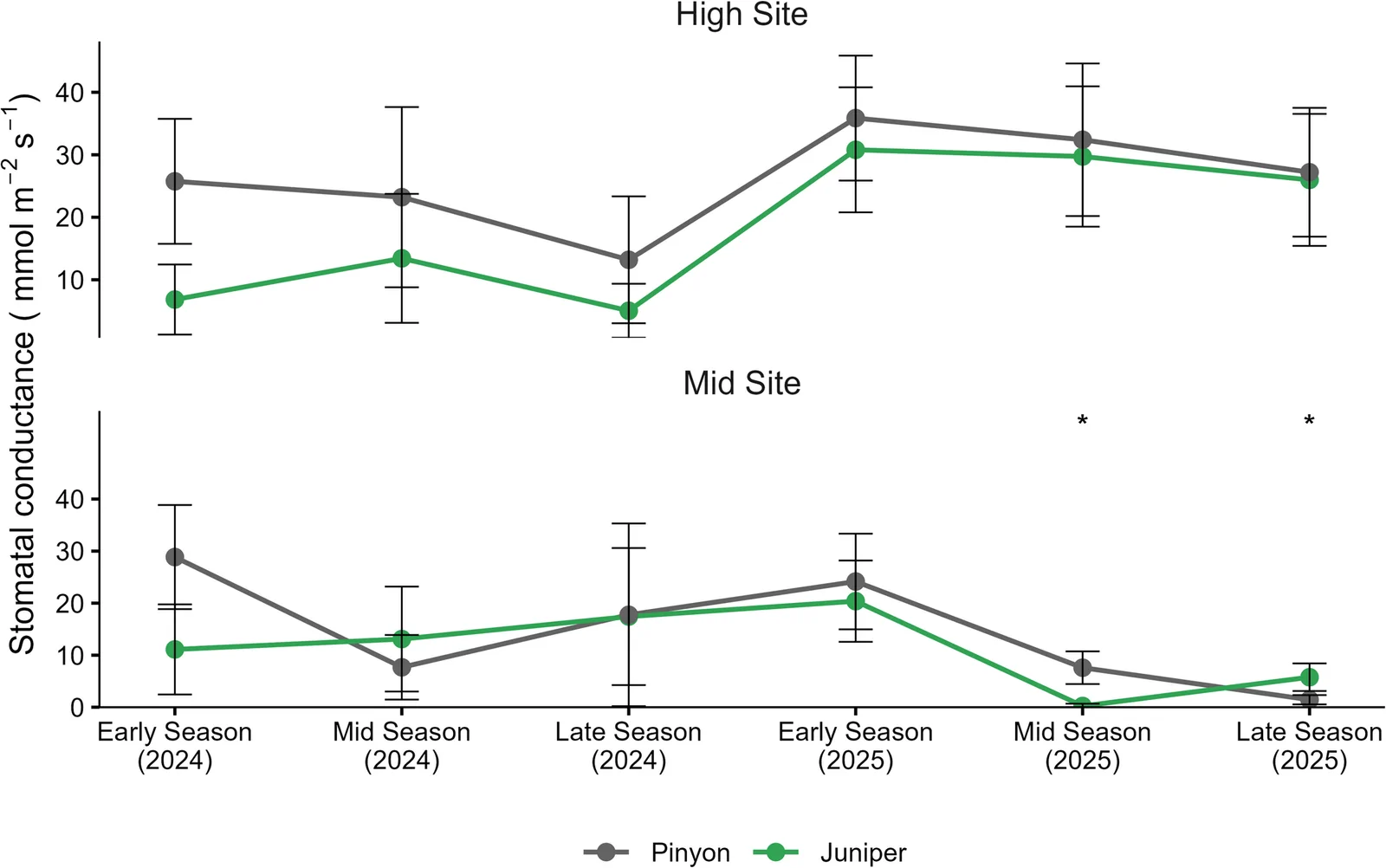
Stomatal conductance for pinyon and juniper during early, mid and late seasons in 2024 (wet) and 2025 (dry) at Mid and High sites. Species differences within each site*season*year combination were tested using pairwise contrasts of estimated marginal means on the log-transformed scale with Holm adjustment. Asterisks indicate significant differences between species (*p* < 0.05)

**Stomatal Conductance.** In the wet year (2024), pinyon showed marginally higher stomatal conductance than juniper (F₁,₂₄ = 4.23, *p* = 0.051). There were no significant effects of season (*p* = 0.74) or site (*p* = 0.56), and no interaction terms were significant (*p* > 0.13). After averaging across site and season, pinyon had 67% higher stomatal conductance than juniper (pinyon: juniper ratio = 1.67, 95% CI = 1.01–2.76, *p* = 0.044; Table S3; Fig. 5). In contrast, species differences were not significant in the dry year (2025; species main effect: F₁,₉₄ = 1.41, *p* = 0.24; Table S4). Instead, stomatal conductance varied strongly with season (F₂,₉₄ = 12.26, *p* < 0.001) and site (F₁,₉₄ = 64.42, *p* < 0.001; Table S3). Effect of species varied among seasons (species × season; *p* = 0.008), seasonal patterns differed across sites (season × site; *p* < 0.001).

**Fig. 6:**
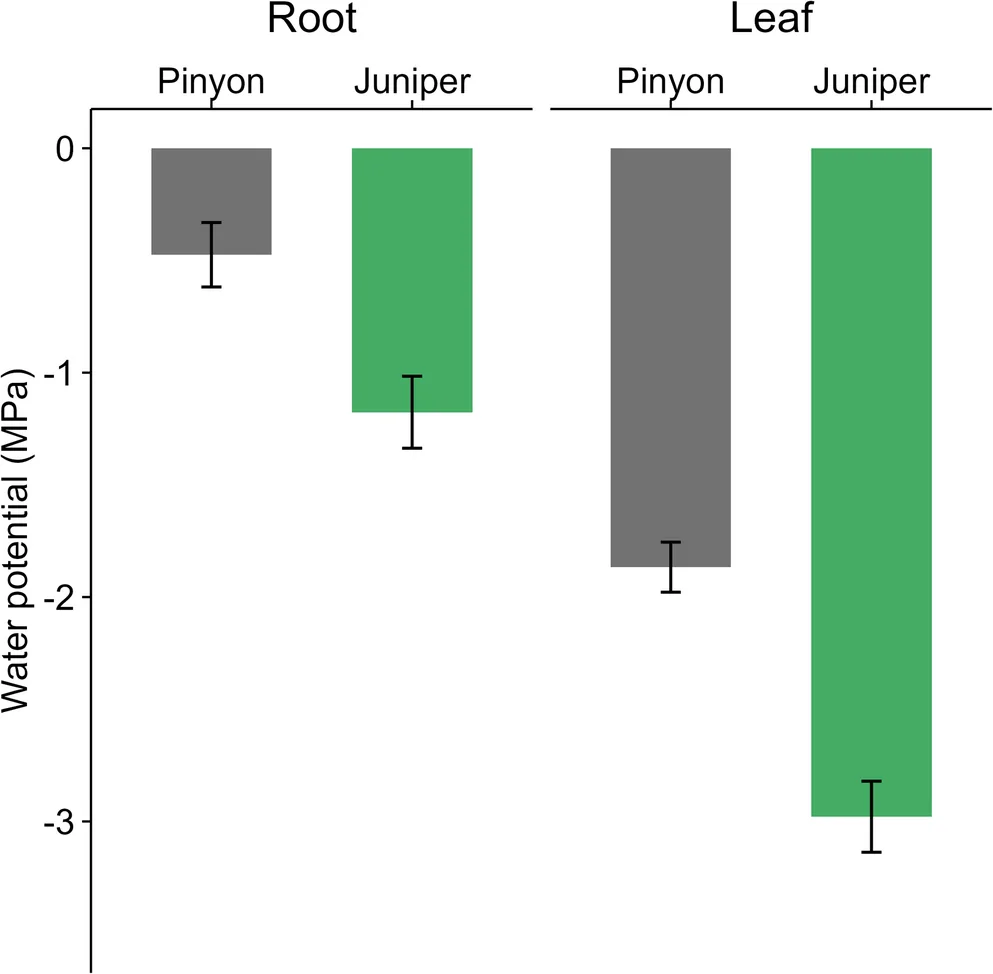
Daytime root and leaf water potential (mean ± SE) for pinyon and juniper. Pinyon leaf and root water potentials were greater than juniper (*P* < 0.005). Error derived from 34 leaf samples and 22 root samples

**Water potential.** Daytime root water potential was higher in pinyon (− 0.48 ± 0.14 MPa) than in juniper (− 1.18 ± 0.16 MPa; Welch’s t-test, t_1,20_ = 3.26, *p* = 0.004; Fig. 6). Leaf water potential was also higher for pinyon (− 1.87 ± 0.11 MPa) than for juniper (− 2.98 ± 0.16 MPa; Welch’s t-test, t_1,30_ = 5.49, *p* < 0.001).

## Discussion

**Fig. 7:**
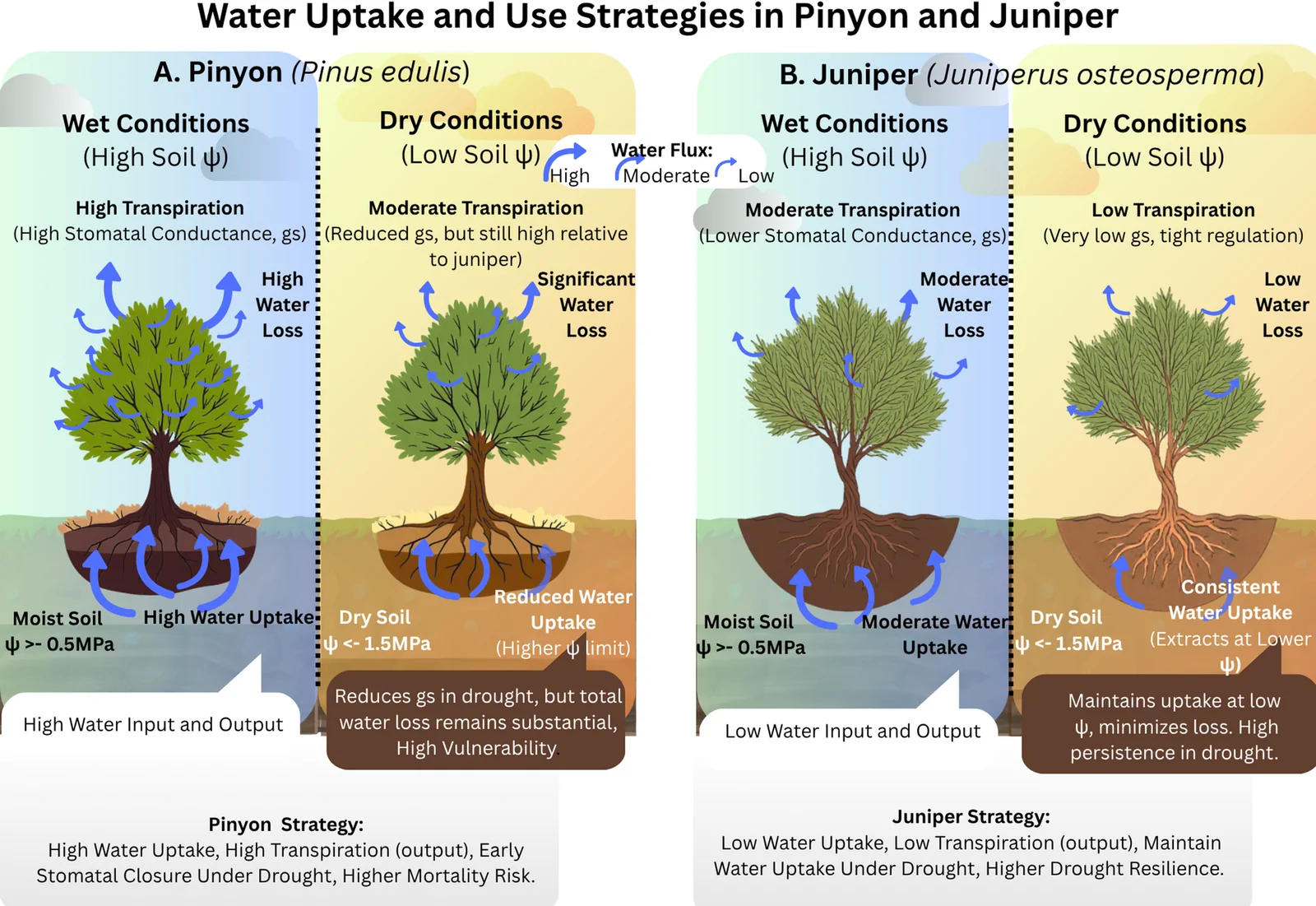
Summary of pinyon and juniper water uptake strategies

Contrary to our predictions, pinyon root distributions absorbed more water than juniper root distributions. Pinyon changed the vertical distribution of roots from month to month in ways that provided 12% more water than juniper. Pinyon decline, therefore, cannot be explained because pinyon roots absorb less water than juniper. Instead, it is likely that, during drought, costly pinyon root foraging strategies have not produced enough water inputs to compensate for rapid pinyon water ‘outputs’ and high carbon costs associated with root foraging (Fig. 7; Wang et al. 2024). Consistent with this idea, we found that pinyon lost more water through its leaves than juniper (Kannenberg et al. 2021; Ghannoum et al. 2025). Our results suggest that pinyon uses ‘fast’ belowground and aboveground water use strategies relative to juniper (Limousin et al. 2015; Aranda et al. 2024). Here, “fast” refers to strategies that move water more quickly through roots and leaves than ‘slow’ strategies. Fast belowground traits (i.e., foraging or changing root distributions from month-to-month) provided greater water inputs, but these greater inputs did not appear to be sufficient to compensate for greater aboveground water ‘outputs’.

*Belowground inputs.* We predicted that pinyon would have (a) shallow, and (b) static root distributions that would (c) absorb less water than juniper (Illuminati et al. 2022; Kulmatiski 2024). Contrary to this prediction, (a) there was no difference in mean rooting depths between pinyon and juniper, (b) pinyon root distributions changed more from month to month than juniper (i.e., pinyon had a more dynamic or plastic rooting strategy), and (c) this plastic rooting strategy absorbed more water than juniper roots over time. Root plasticity was assessed with the CV of tracer uptake across sampling months. The CV of tracer uptake was greater for pinyon than juniper throughout the soil profile (Fig. 4). Pinyon tracer uptake changed by 25% from month to month, while juniper tracer uptake changed by about 16% from month to month. When these tracer uptake profiles were used in a soil water model to estimate water uptake over time, the greater month-to-month variation in pinyon tracer uptake resulted in pinyon absorbing 12% more water than juniper across the two years of the study. This provides clear support for the idea that pinyon actively foraged for water. In other words, pinyon increased soil water uptake by ‘moving’ roots toward moist soils (i.e., a ‘fast’ belowground growth strategy; Plaut et al. 2012; Clifford et al. 2013; Guérin et al. 2018).

Rooting plasticity can occur through several mechanisms, and most require more energy than maintaining a static root distribution. Over hours to days, plants can increase aquaporin activity or abundance to increase water uptake (Gambetta et al. 2017). Plants can also change cellular water potentials to increase or decrease water uptake (Blum et al. 2017; Zivcak et al. 2016). Over longer periods of days to weeks, plants can grow new fine roots and over even longer periods, coarse roots. Each of these mechanisms requires metabolism. Inasmuch, greater root plasticity in pinyon can be assumed to be associated with greater carbon costs (West et al. 2007; Anderegg et al. 2016). Pinyon, therefore, appeared to maintain a high carbon input / high water output or ‘fast’ belowground root strategy.

Foraging ‘choices’ made by pinyon were complex and perhaps easier to understand in relation to juniper foraging ‘choices’. Juniper tracer uptake profiles were fairly consistent among months. Maximum juniper tracer uptake consistently occurred at either 5 or 30 cm at a site (Figs. 1 and 2). It is likely that low tissue water potentials allowed juniper to extract water that was not available to other species decreasing the need for juniper to ‘move’ roots to ‘find’ transient water (Fig. 5; West et al. 2008; Limousin et al. 2013; Hudson et al. 2018). When we performed a set of Hydrus simulations to estimate how much more water a plant with a minimum water potential of -4.5 MPa (i.e., juniper) could absorb relative to a plant with a minimum water potential of -2.5 MPa (i.e., pinyon), we found that lower water potentials would allow juniper to absorb 12% more water than pinyon (Mackay et al. 2015; Hudson et al. 2018; Aranda et al. 2024). We suggest that pinyon compensated for this disadvantage by changing root distributions to follow available water pulses (West et al. 2007; Meinzer et al. 2014; Hudson et al. 2018). Consistent with this idea, maximum pinyon tracer uptake occurred in 1, 5, 15, 30, and 90 cm in different sites and months.

Pinyon was estimated to have 12% water uptake advantage due to changing root distributions and a 12% water uptake disadvantage due to higher water potentials. As a result, these opposing traits produced no net water uptake advantage (~ 0%) for pinyon. Tracer uptake data produced similar results. The deuterium concentrations in pinyon transpiration water (144‰ or 1.78e^− 4^ deuterium per hydrogen) were only 2% greater than juniper (122‰ or 1.75e^− 4^ deuterium per hydrogen). We are not arguing that this is a large or biologically important water uptake advantage, rather, that tracer uptake measured directly and water uptake estimated using a soil water flow model and proportional tracer uptake data provided somewhat independent and similar estimates of water uptake.

There are many potential sources of variation in our analyses that make the similarity in tracer and water uptake values notable. Variation in tracer uptake data, for example, is derived from variation among sampled individual trees as well as locations within trees. The water flow model is deterministic, but is likely to vary from tracer uptake data because weather and soil patterns can vary widely at small scales. For example, some sampled trees were located on shallow soils while others were located on deep soils and the soil water flow model assumed the same 100 cm soils in all plots. Similarly, weather can vary widely over just a few kilometers at the study site due to small storm cells. So, while the soil water model provided important insight into water uptake between the six injection dates, there were reasons that water flow would be different for sampled trees than estimated by the model.

The 0–2% water and tracer uptake advantage estimated for pinyon was small relative to pinyon water ‘outputs’. Pinyon stomatal conductance was 67% greater than juniper in the wet year and 25% greater than juniper across both study years suggesting that pinyon requires more water than juniper. Pinyon, therefore, may have declined in the past 25 years because there has not been sufficient water for rapid pinyon growth belowground and aboveground (Breshears et al. 2009; Pangle et al. 2015).

*Fast versus slow belowground and aboveground strategies.* Results suggest that pinyon relies on a ‘grow fast in good conditions’ strategy that has been unsuccessful during the hotter and drier conditions that have dominated much of the western United States in recent decades (Mueller et al. 2005; Shaw 2008; Barger et al. 2009). This idea is supported by the fact that pinyon water uptake was more dynamic and greater than juniper in the wet but not the dry year (Figs. 2 and 3) and that pinyon stomatal conductance was greater than juniper in the early season and in the High (wetter) site (Fig. 5). In contrast, stomatal conductance was similar for pinyon and juniper during the dry year (Plaut et al. 2012; Pangle et al. 2015). These results indicate that pinyon increases physiological activity when soil moisture is abundant, but that juniper can maintain growth at lower water availability (Limousin et al. 2013; Kraklow et al. 2025). These ‘fast’ belowground and aboveground strategies are likely to allow rapid growth when soil water is available, but will lead to cavitation and high carbon costs in pinyon in the hot and dry conditions (Trugman et al. 2018). In contrast, juniper relies on a slower ‘stress-tolerator’ approach that has allowed it to grow when water potentials are too low for other species including grasses and shrubs (Willson et al. 2008; Peterman et al. 2013). Overall, this integrative approach links belowground water uptake with aboveground water use, providing a mechanistic explanation for previously described species differences.

Pinyon and juniper grow in semiarid conditions with roughly 250 to 500 mm mean annual precipitation. Deep percolation (below 90 cm) with this amount of precipitation is likely to occur only in rocky soils. Our soil cores and soil water flow modeling support this conclusion. Our soil water flow model indicated that 13% of soil water uptake occurred below 75 cm in the wet year at the low site. This is consistent with global root biomass surveys which indicate that 50 to 95% of root biomass in semiarid systems occurs at 100 to 190 cm (Schenk and Jackson 2002) and that deep roots are likely to be more important for mineral uptake and maintenance water rather than for total water uptake (Bachofen et al. 2024; McCulley et al. 2004; Ryel et al. 2008). Very deep roots that access the aquifer are unlikely at this site. The nearest data (~ 50 km away) indicates a water table depth of 50 m. For these reasons, we expect that our inference to the top meter of soil represented the vast majority of water uptake.

*Conclusions.* We suggest that pinyon has declined not only because pinyon outputs are greater than juniper outputs, but because ‘fast’ water use strategies are disfavored during drought (Trugman et al. 2018). Our results indicate that pinyon uses costly belowground strategies to supply as much water as possible. This is an important finding because this is a higher risk strategy than would be understood from aboveground measurements alone. In other words, pinyon appears to be investing in active foraging to maintain high water output even in conditions that threaten pinyon mortality. An alternative, more conservative strategy, would be to use carbon invested in root foraging to protect or repair xylem or slow leaf growth. Fast water uptake and use is likely to result in faster transition from rapid growth to mortality than would be expected from aboveground traits alone. This suggests that pinyon will be faster to die during droughts and faster to recover during wet periods than expected from aboveground traits alone.

Results help fill a gap in understanding of pinyon and juniper ecohydrology (Plaut et al. 2012; Koepke and Kolb 2013). Previous research described pinyon and juniper water ‘outputs’, but little was known about water ‘inputs’. Our results suggest that pinyon uses ‘fast’ growth strategies both belowground and aboveground relative to juniper. These ‘fast’ strategies provide a small ‘input’ advantage that is smaller than the associated ‘output’ disadvantage (Breshears et al. 2005; Koepke and Kolb 2013; Pangle et al. 2015). Given continued warming and drying projected for the future, our results suggest that juniper may continue to expand while pinyon continues to decline (Floyd et al. 2009; Redmond et al. 2023).

## Supplementary Information

Below is the link to the electronic supplementary material.

Supplementary Material 1

## Data Availability

All data and programming used in this research will be made publicly available in a permanent online repository upon acceptance.

## Code Availability

All programming used in this research will be made publicly available in a permanent online repository upon acceptance.
