# Supplementary material for: ‘Fast’ and ‘slow’ water handling strategies explain pinyon pine decline and juniper expansion
Source: Oecologia. 2026 Jul 20;208(8):95. doi: 10.1007/s00442-026-05929-y (PMC13385175; doi:10.1007/s00442-026-05929-y)
Supplement: Supplementary file 1 — Supplementary Material 1 [file 442_2026_5929_MOESM1_ESM.docx]

**Supplementary data

Table S1.** Soil hydraulic and root uptake parameters used in HYDRUS-1D simulations.

| \| **Parameter** \| **Value(s)** \| \| --- \| --- \| \| Time units \| Days \| \| Materials \| 0-3, 3-15, 15-30, 30-60,60-100 cm \| \| Hydraulic Model \| van Genuchten – Mualem \| \| Hysteresis \| No hysteresis \| \| Root water uptake model \| Feddes \| \| Critical Stress index for Water Uptake \| 1 \| \| Feddes parameters (P0, POpt, P2H, P2L, P3, r2L) \| –10, –25, –1500, –1500, –25000, 0.01 \| \| Feddes (r2H) \| 0.2, 0.3, 0.4 for early, mid, late season \| \| HCitA \| 10000 \| \| Crop height \| 50 cm \| \| LAI \| 0.5, 0.6, 0.7 for early, mid, late season \| \| Upper boundary \| Atmospheric BC with surface Run off \| \| Lower boundary \| Free drainage \| \| Radiation \| Solar Radiation \| \| Radiation Extinction \| 0.5 \| \| Cloudiness \| Solar Radiation \| \| Crop Data \| constant \| |
| --- | --- | --- | --- | --- | --- | --- | --- | --- | --- | --- | --- | --- | --- | --- | --- | --- | --- | --- | --- | --- | --- | --- | --- | --- | --- | --- | --- | --- | --- | --- | --- | --- | --- | --- | --- | --- |

**Fig. S1**. Observed versus predicted volumetric soil moisture at a) 10 cm, b) 25 cm, and c) 50 cm. Observed data collected with volumetric soil sensors at the low site. Predicted data calculated in Hydrus 1D.

Proportion uptake by depth values are used instead of raw values larger plants dilute tracer signals more than smaller plants. For example, in May, 3% of total tracer uptake across soil depths may have occurred in the 1 cm depth injection plots. This value was then converted to a per cm value by dividing by the depth increment each injection represents. For example, the 1 cm injections are assumed to represent water uptake from 0-3 cm, so the 3% uptake from 1 cm plots is reported as 3% / 3 cm = 1% uptake per cm soil depth. More specifically, for each plot, the proportional uptake at each depth was calculated as

$$P_{n}= \frac{S_{n}}{\sum_{i=1}^{k} S_{i}}$$

Where $S_{n}$is the tracer value at depth *n* for a given plot, and $\sum_{i=1}^{k} S_{i}$is the sum of tracer values across all depths for that same plot. These calculations produce a proportion uptake value for each site × month × depth × species × replicate combination.

**Table S1**. Akaike Information Criterion (AIC) comparison between beta regression generalized additive models (GAMs) with a logit link function, including global models (shared depth smooth across species) and root models (species-specific depth smooths), evaluating tracer uptake patterns of pinyon and juniper across seasons and sites in 2024 (wet year) and 2025 (dry year).

| **Site** | **Season** | **2024 AIC (Global)** | **2024 AIC (Root)** | **ΔAIC (Global − Root)** | **2025 AIC (Global)** | | **2025 AIC (Root)** | **ΔAIC (Global − Root)** | **df**  **(Global)** | **df (Root)** |
| --- | --- | --- | --- | --- | --- | --- | --- | --- | --- | --- |
| Mid | Early | -769 | -772 | 3 | -1486 | -1550 | | 64 | 3 | 8 |
| Mid | Mid | -678 | -685 | 7 | -1505 | -1568 | | 63 | 3 | 8 |
| Mid | Late | -780 | -782 | 2 | -1417 | -1480 | | 63 | 3 | 8 |
| High | Early | -780 | -429 | 351 | -1549 | -1589 | | 40 | 4 | 8 |
| High | Mid | -384 | -390 | 6 | -1557 | -1600 | | 43 | 4 | 8 |
| High | Late | -480 | -481 | 1 | -1358 | -1413 | | 55 | 4 | 8 |


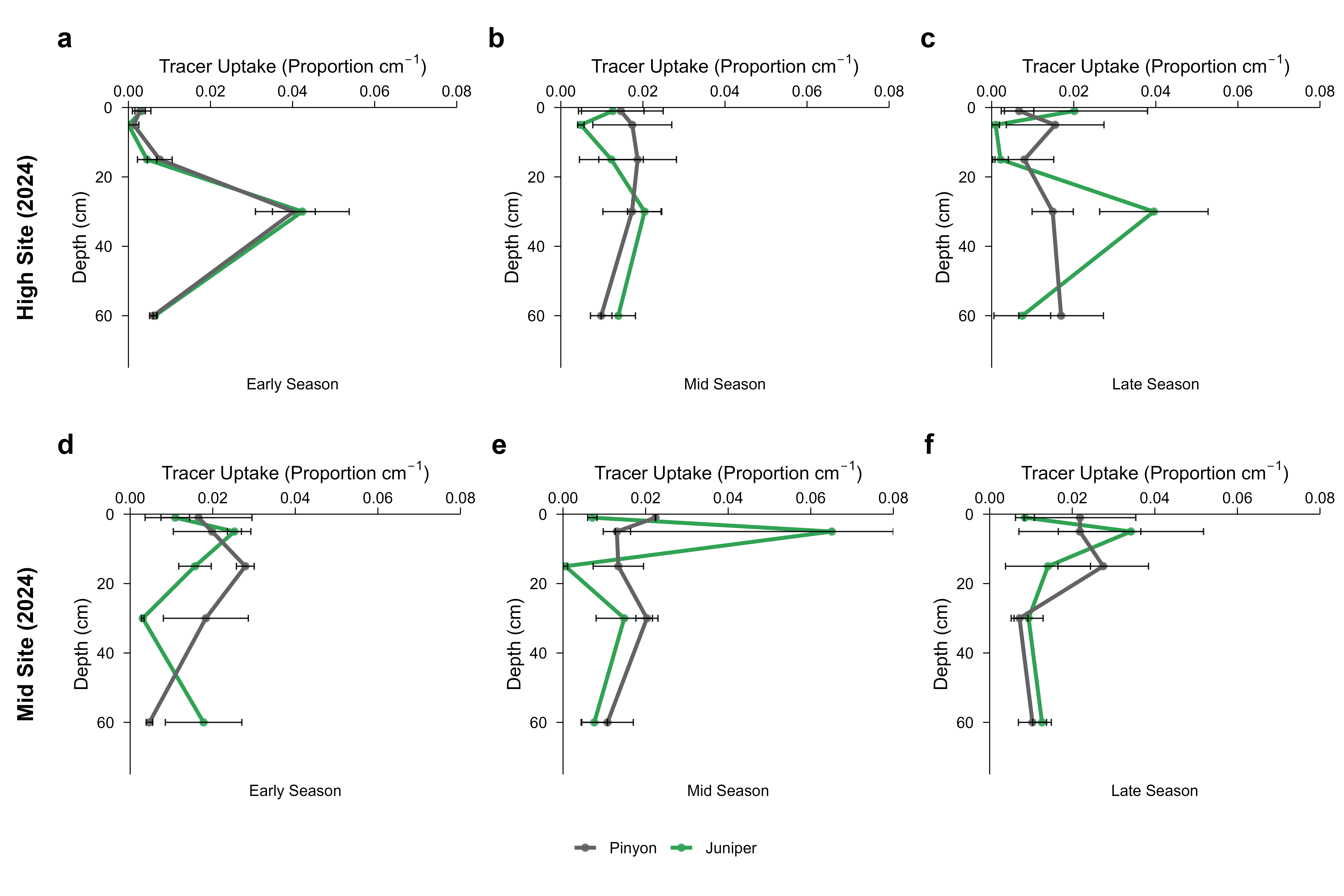


**Figure S2.** Tracer uptake (proportion cm⁻¹) for pinyon and juniper during the wet year (2024) at the high site (panels a–c) and the mid site (panels d–f) across early, mid, and late seasons. Values represent mean tracer uptake (cm cm⁻¹) ± SE.

**
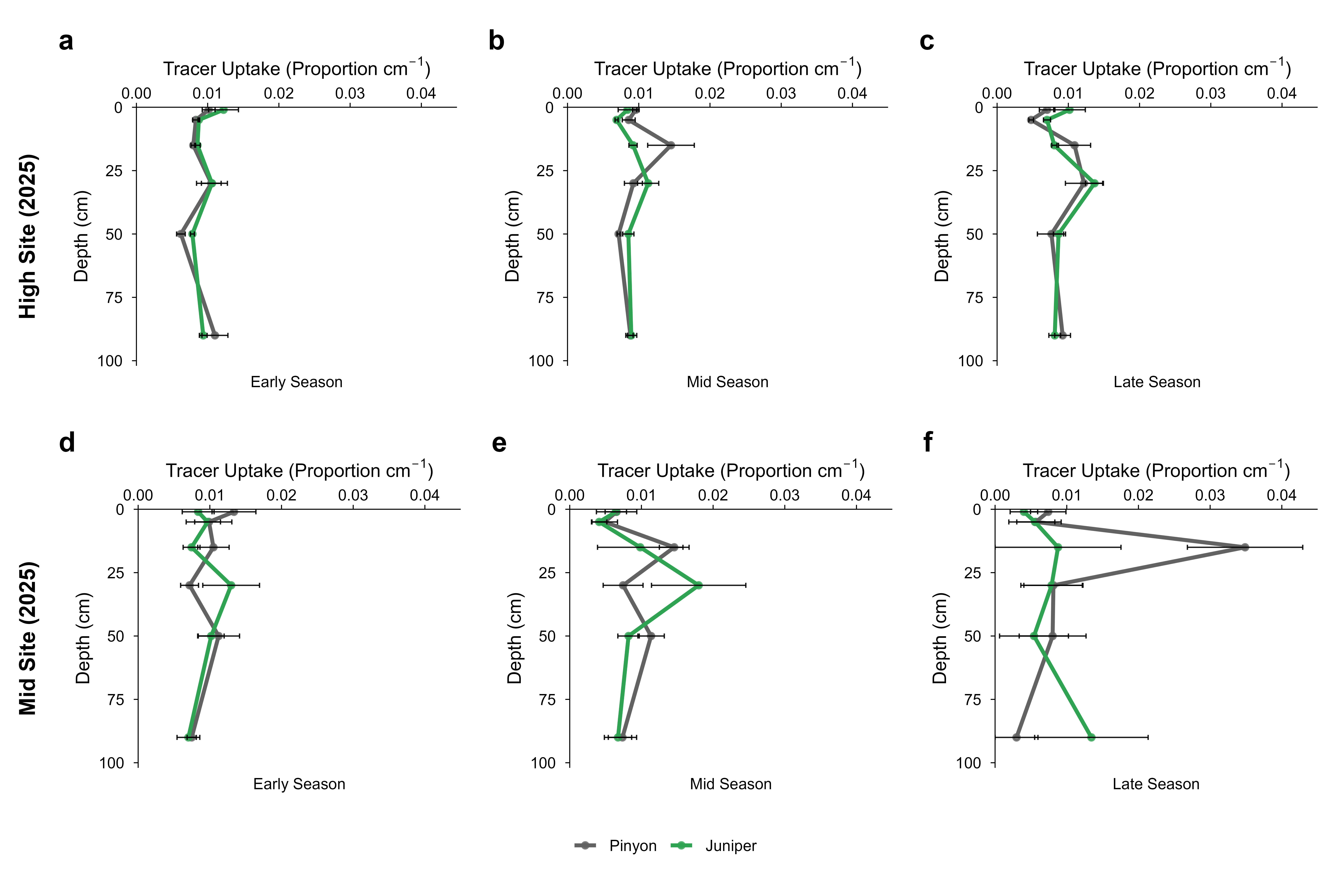
**

**Figure S3.** Tracer uptake (proportion cm⁻¹) for pinyon and juniper during the dry year (2025) at the high site (panels a–c) and the mid site (panels d–f) across early, mid, and late seasons. Values represent mean tracer uptake (cm cm⁻¹) ± SE.


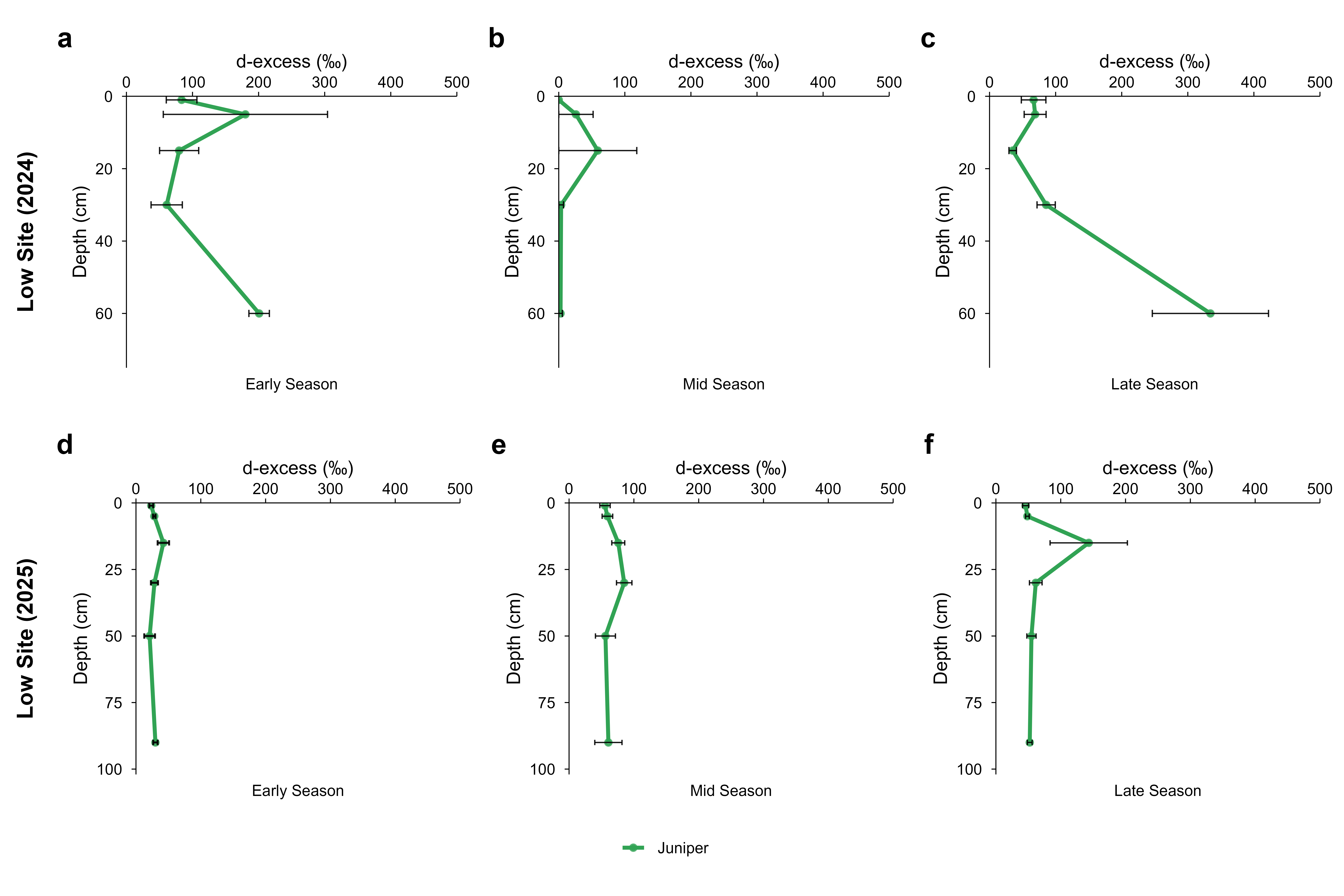


**Figure S4.** D-excess for juniper during the wet year (2024) at the low site (panels a–c) and dry year (2025) the low site (panels d–f) across early, mid, and late seasons. Values represent mean tracer uptake (cm cm⁻¹) ± SE.


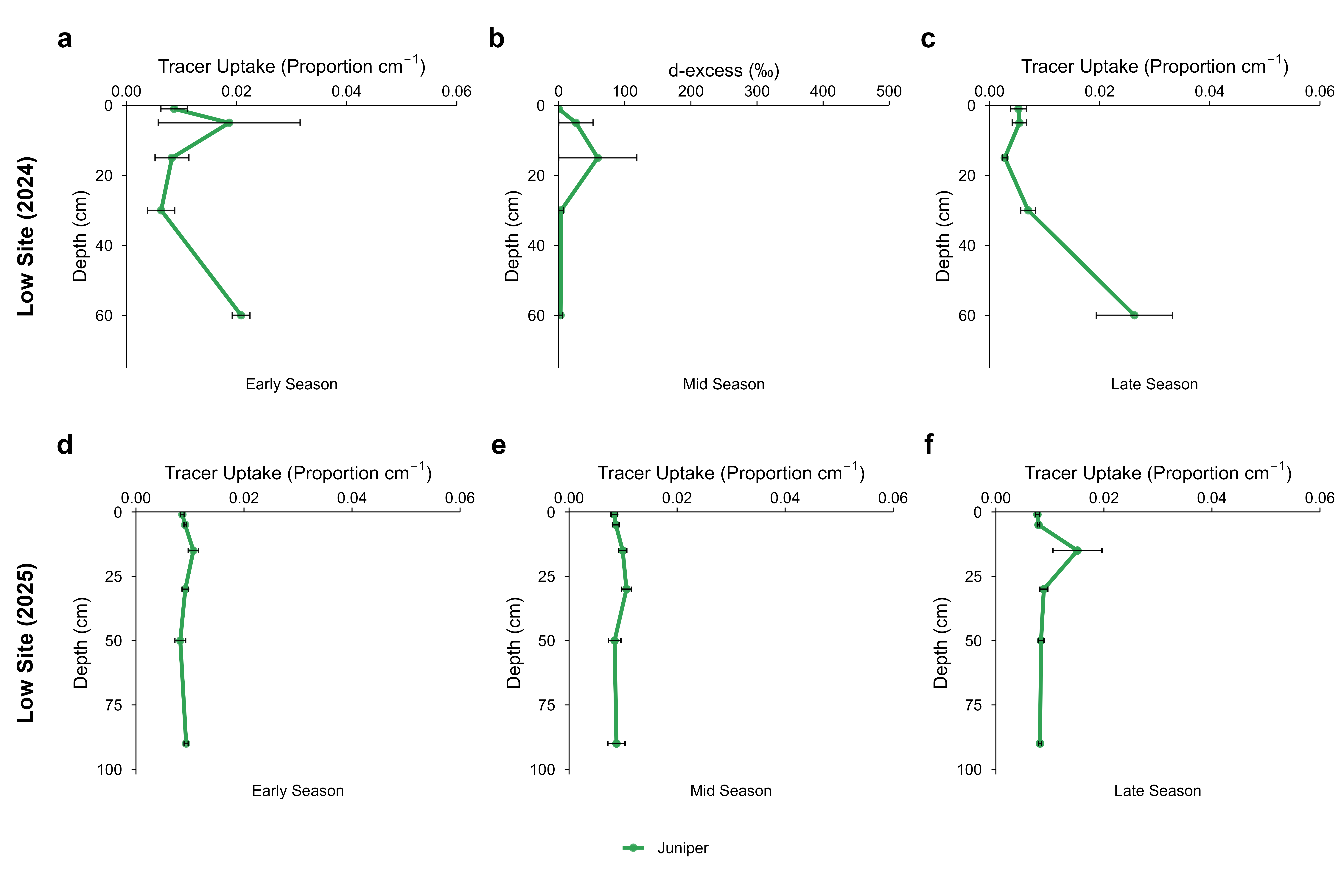


**Figure S5.** Tracer uptake (proportion cm⁻¹) for juniper during the wet year (2024) at the low site (panels a–c) and dry year (2025) the low site (panels d–f) across early, mid, and late seasons. Values represent mean tracer uptake (cm cm⁻¹) ± SE.

**Table S3.** Mean (± SD) sand%, silt%, clay% and soil bulk density by depth and site.

| **Site** | **Depth (cm)** | **Sand (%)** | **Silt (%)** | **Clay (%)** | **Bulk density (g cm⁻³)** | **n** |
| --- | --- | --- | --- | --- | --- | --- |
| Low | 0–15 | 81.3 ± 8.3 | 13.3 ± 9.2 | 5.3 ± 2.3 | 1.44 ± 0.06 | 3 |
| Low | 15–30 | 78.3 ± 10.0 | 12.3 ± 7.8 | 9.3 ± 2.3 | 1.03 ± 0.09 | 3 |
| Low | 30–45 | 71.3 ± 8.1 | 18.0 ± 6.0 | 10.7 ± 2.3 | 0.95 ± 0.08 | 3 |
| Low | 45–60 | 67.7 ± 2.1 | 21.0 ± 1.7 | 11.3 ± 3.1 | 1.14 ± 0.08 | 3 |
| Low | 60–75 | 71.0 ± 8.5 | 17.0 ± 6.1 | 12.0 ± 3.5 | 0.95 ± 0.08 | 3 |
| Mid | 0–15 | 70.0 ± 11.3 | 25.0 ± 9.9 | 5.0 ± 1.4 | 1.32 ± 0.01 | 2 |
| Mid | 15–30 | 67.0 ± 4.2 | 16.0 ± 8.5 | 17.0 ± 4.2 | 1.27 ± 0.11 | 2 |
| Mid | 30–45 | 53.5 ± 0.7 | 27.5 ± 2.1 | 19.0 ± 1.4 | 1.00 ± 0.06 | 2 |
| Mid | 45–60 | 57.0 ± 1.4 | 23.0 ± 1.4 | 20.0 ± 2.8 | 1.04 ± 0.03 | 2 |
| Mid | 60–75 | 58.0 ± 2.8 | 22.0 ± 2.8 | 20.0 ± 0.0 | 1.18 ± 0.30 | 2 |
| High | 0–15 | 70.3 ± 7.5 | 20.3 ± 4.5 | 9.3 ± 3.1 | 1.06 ± 0.06 | 3 |
| High | 15–30 | 74.0 ± 12.1 | 15.3 ± 11.6 | 10.7 ± 1.2 | 0.98 ± 0.09 | 3 |
| High | 30–45 | 73.3 ± 2.3 | 18.0 ± 4.0 | 8.7 ± 2.3 | 0.88 ± 0.06 | 3 |
| High | 45–60 | 73.0 ± 3.6 | 17.0 ± 3.6 | 10.0 ± 2.0 | 0.96 ± 0.09 | 3 |
| High | 60–75 | 74.5 ± 4.9 | 15.5 ± 2.1 | 10.0 ± 2.8 | 1.12 ± 0.01 | 2 |

| **Table S3.** Type III ANOVA results for log-transformed stomatal conductance (2024; n = 30 observations).   \| **Effect** \| **df** \| **Sum of Squares** \| **F value** \| **p value** \| \| --- \| --- \| --- \| --- \| --- \| \| Species \| 1 \| 2.221 \| 4.23 \| 0.051 \| \| Season \| 2 \| 0.313 \| 0.30 \| 0.745 \| \| Site \| 1 \| 0.185 \| 0.35 \| 0.558 \| \| Species × Season \| 2 \| 1.705 \| 1.62 \| 0.218 \| \| Species × Site \| 1 \| 1.123 \| 2.14 \| 0.157 \| \| Season × Site \| 2 \| 2.251 \| 2.14 \| 0.139 \| \| Species × Season × Site \| 2 \| 0.189 \| 0.18 \| 0.836 \| \| Residuals \| 24 \| 12.598 \|  \|  \| |
| --- | --- | --- | --- | --- | --- | --- | --- | --- | --- | --- | --- | --- | --- | --- | --- | --- | --- | --- | --- | --- | --- | --- | --- | --- | --- | --- | --- | --- | --- | --- | --- | --- | --- | --- | --- | --- | --- | --- | --- | --- | --- | --- | --- | --- | --- |

**Table S4**. Type III ANOVA results for log-transformed stomatal conductance (2025; n = 100 observations).

| **Effect** | **df** | **Sum of Squares** | **F value** | **p value** |
| --- | --- | --- | --- | --- |
| Species | 1 | 1.32 | 1.41 | 0.238 |
| Season | 2 | 22.98 | 12.26 | <0.001 |
| Site | 1 | 60.36 | 64.42 | <0.001 |
| Species × Season | 2 | 9.62 | 5.14 | 0.0076 |
| Species × Site | 1 | 0.46 | 0.49 | 0.486 |
| Season × Site | 2 | 17.55 | 9.36 | <0.001 |
| Species × Season × Site | 2 | 9.29 | 4.96 | 0.0090 |
| Residuals | 94 | 88.08 |  |  |
